# Physiologically Based Pharmacokinetic Modeling of Nanoparticle Biodistribution: A Review of Existing Models, Simulation Software, and Data Analysis Tools

**DOI:** 10.3390/ijms232012560

**Published:** 2022-10-19

**Authors:** Elena O. Kutumova, Ilya R. Akberdin, Ilya N. Kiselev, Ruslan N. Sharipov, Vera S. Egorova, Anastasiia O. Syrocheva, Alessandro Parodi, Andrey A. Zamyatnin, Fedor A. Kolpakov

**Affiliations:** 1 Scientific Center for Information Technologies and Artificial Intelligence, Sirius University of Science and Technology, 354340 Sochi, Russia; 2 Federal Research Center for Information and Computational Technologies, 630090 Novosibirsk, Russia; 3 BIOSOFT.RU, Ltd., 630058 Novosibirsk, Russia; 4 Department of Natural Sciences, Novosibirsk State University, 630090 Novosibirsk, Russia; 5 Specialized Educational Scientific Center, Novosibirsk State University, 630090 Novosibirsk, Russia; 6 Scientific Center for Translational Medicine, Sirius University of Science and Technology, 354340 Sochi, Russia; 7 Institute of Molecular Medicine, Sechenov First Moscow State Medical University, 119991 Moscow, Russia; 8 Belozersky Institute of Physico-Chemical Biology, Lomonosov Moscow State University, 119992 Moscow, Russia

**Keywords:** nanoparticles, physiologically based pharmacokinetic modeling, simulation software, BioUML

## Abstract

Cancer treatment and pharmaceutical development require targeted treatment and less toxic therapeutic intervention to achieve real progress against this disease. In this scenario, nanomedicine emerged as a reliable tool to improve drug pharmacokinetics and to translate to the clinical biologics based on large molecules. However, the ability of our body to recognize foreign objects together with carrier transport heterogeneity derived from the combination of particle physical and chemical properties, payload and surface modification, make the designing of effective carriers very difficult. In this scenario, physiologically based pharmacokinetic modeling can help to design the particles and eventually predict their ability to reach the target and treat the tumor. This effort is performed by scientists with specific expertise and skills and familiarity with artificial intelligence tools such as advanced software that are not usually in the “cords” of traditional medical or material researchers. The goal of this review was to highlight the advantages that computational modeling could provide to nanomedicine and bring together scientists with different background by portraying in the most simple way the work of computational developers through the description of the tools that they use to predict nanoparticle transport and tumor targeting in our body.

## 1. Introduction

Cancer disease still represents the leading cause of death worldwide [1], and its treatment, still largely based on cytostatic approaches [2], can be very difficult, especially in the presence of metastases [3]. Targeted therapies based on nanodelivery systems polarized the attention of cancer researchers after the enhanced permeability and retention effect (EPR) was discovered in 1986 [4]. The first goal of nanomedicine was to develop targeted systems to accumulate the therapeutic payload only in the cancer lesion [5], sparing off-site organs to develop safer therapeutic regimens, and administer less active principle with improved therapeutic performances [6,7]. Two major delivery approaches have been developed imparting nanocarriers with passive and active targeting properties. Passive targeting is achieved by conferring to nanoparticles (NPs) the properties of long-term circulation and the ability to accumulate in the cancer lesion through EPR [8]. Active targeting strategies rely on the affinity between the surface of the particles, usually decorated with small molecules that recognize specific antigens or receptors over-expressed on the surface of cancer cells. In this case, the carriers still rely on passive extravasation to interact with cancer cells [8], but their strong interaction with the cell membrane can promote their internalization [9]. In addition, some surface modifications can promote the passage of NPs through the endothelial wall or the development of strategies based on the Trojan horse approaches, when particles are transported to the tumor by infiltration of immune cells [6]. In a very simplified way, we can describe the nanomedicine pipeline as comprising carrier synthesis, drug encapsulation and release, NP targeting, cellular internalization, and drug delivery, as well as the impact of NP on cells and tissues [10]. However, the complexity of this process is significantly higher. There is no limitation on the kind of therapeutic payload that can be encapsulated, including (but not limited to) small molecules [11,12], new generations of biologics (nucleic acids [13] and peptides [14]), as well as molecules for the improvement of modern radiotherapy [15] and diagnostics [16]. All these payloads are characterized by their own physical and chemical properties and need to be investigated separately. In addition, the carrier per se can also be a treatment, providing a means for the development of theranostics [17] and a trigger for thermal ablation [18] and photodynamic therapy [19]. There are also no limitations in terms of the materials researched to create the carriers. To this end, biological molecules such as lipids, nucleic acids, proteins, and sugars have been extensively studied, in addition to natural (e.g., chitin) and synthetic (e.g., polylactic acid) polymers and inorganic materials (iron, gold, silver, platinum, and silica) [20,21]. To add to this variety, it is worth mentioning the generation of hybrid delivery platforms with improved delivery and biocompatibility properties [22,23], surface modifications, as well as physical (size, shape, surface charge) and chemical properties (degradation rate) specific for every carrier. Each individual combination and variation in these elements can have a major impact on particle distribution and tumor targeting. As a result, in terms of pharmacokinetics benefit, it can be very difficult to develop a successful universal carrier and each nanoformulation requires intensive, expensive, and time consuming in vitro and in vivo experimental validation. A way to speed up this process is to use modern modeling and artificial intelligence approaches to obtain at least approximate indications on the fate of carriers and their payload, as well as their effectiveness and potential toxicity. Here, modern physiologically based pharmacokinetic (PBPK) models can provide the tools to describe and predict the systemic localization, target exposure, efficacy, and toxicity of various nanotherapeutic agents [24,25,26]. In consideration of these important benefits, their development and use is constantly expanding [26], providing important in silico tools to bridge drug properties and in vivo PK behaviors during drug development [27]. In recent years, artificial intelligence and machine learning approaches have been increasingly applied to PBPK modeling for nanomaterials [28,29,30,31]. However, further progress in this area seems to be rather difficult, as it requires specialists to have knowledge from fundamentally distant fields of knowledge: materials science, biology, and informatics. We believe that a detailed description of the methods and tools that the modeler uses to simulate NP localization can help the reader better understand the published results, the benefits of the selected modeling approaches, and the importance of the results for nanomedicine. These tools are software with different characteristics and levels of complexity, whose developers focused on creating a convenient user interface to facilitate the use of these programs by specialists who do not have deep knowledge in this area. In this review, we strove to describe in the most accessible language the activities of the developer in creating PBPK models of NPs. Moreover, we described the computational tools (software) harnessed to build and analyze the PBPK models, highlighting the necessity and time to change the model representation paradigm in this area for greater transparency and reliable reproducibility of the simulation results based on the state-of-the-art system biology standards such as Systems Biology Markup Language (SBML) [32] and Systems Biology Graphical Notation (SBGN) [33]. In this effort, we provided information on their application to predict and characterize tumor targeting and cancer treatment with NPs. The final goal of this review was to solve the lost in translation problem between experimental biomedical research and computational modeling to favor interaction between multidisciplinary scientists. Compared to other reviews in the field [24,25,26,27,34,35], we tried to achieve this goal by presenting the work of a computational scientist through the description of the tools that she/he used to generate PBPK models. We believe that this kind of information could be helpful to a not experienced audience in the field to obtain some background about this kind of research and build new ideas and collaborations. In addition, this article contains the most comprehensive list of currently available PBPK models for nanoparticles (documented in Supplementary Table S1), which might be useful for developers of new models.

## 2. Importance of Mathematical Modeling in Nanomedicine

**Figure 1:**
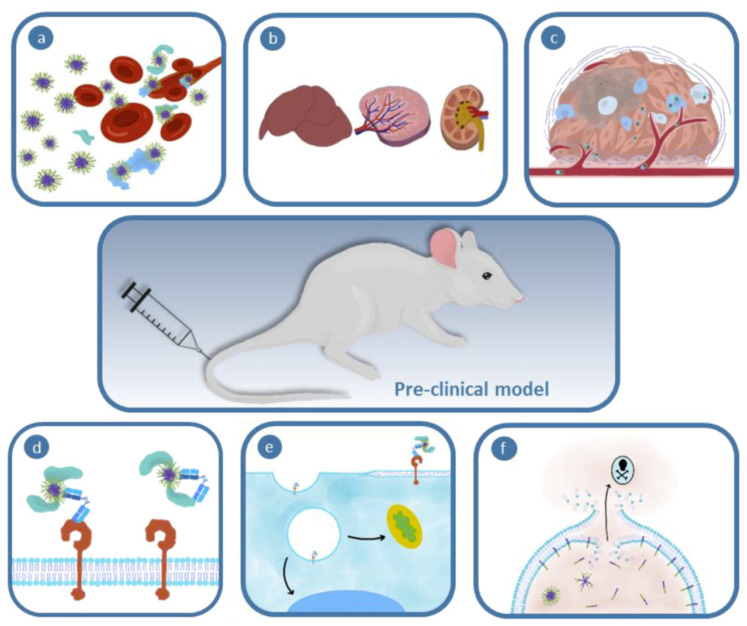
Schematic of the major biological barriers encountered during intravenous treatment including (**a**) interactions with blood components; (**b**) mononuclear phagocytic system organ clearance; (**c**) tumor barriers (i.e., tumor extracellular matrix); (**d**) biochemical barriers; (**e**,**f**) cellular barriers including cell membrane, interaction with cellular receptor, intracellular sorting and sequestration in the endosomal compartment. Figure adapted from Simpson et al. [43].

One of the most important results derived from nanomedicine research has been a better understanding of the behavior of various materials processed at the nanoscale in the biological environment and the generation of nanocarriers with different physical, payload retention, and surface properties. Carriers have been designed with different functions to improve the pharmacokinetic properties of various drugs [36,37] while protecting the therapeutic burden from the biological environment. While the primary goal of nanomedicine development has always been clinical translation, many works highlighted the difficulties and obstacles in the translation of nanocarrier-based therapeutic regimens [38]. Most of these difficulties are because our bodies are very effective in recognizing and isolating foreign bodies [39,40]. Nanomedicine impact to clinics was significantly mitigated by the complexity of our bodies and their ability to regulate therapeutic transport between different compartments, commonly known as biological barriers [41]. Considering that most therapies have been designed for intravenous administration, the path from the point of injection to the cancerous lesion can be hampered by many biological barriers, sometimes limiting tumor targeting to less than 1% [42]. These biological barriers are of physical, cellular, and molecular nature, and they very often appear simultaneously during the NP voyage to the target (Figure 1) [43]. Their action strictly depends on the physical properties of NPs (shape, size, and surface charge), which in the end, can also be determined by the payload. In addition, the surface properties of the particles result in the acquisition of a specific biologic identity because of the absorption of biological molecules [44], eventually determining their final disposition [45]. In this context, computational modeling represents an important tool for determining the interactions of NPs with biological structures and proposing the main characteristics to grant the possibility of simultaneous and sequential passage through biological barriers [41].

Current strategies for tumor treatment include targeting malignant and metastatic components of the tumor microenvironment [12], as well as targeting over-activated metabolic signaling pathways characteristic of cancerous tissue [46]. Mathematical modeling supports the connection of entities of different scales (from blood vessels to surface interaction with biological molecules), which leads to an improved interpretation of specific tumor characteristics [47]. The modeling of such systems actually requires the use of multiscale approaches that can store information transferred between different scales [48]. The development of nanotherapeutics is closely related to computer simulations and many mathematical models have been developed to study various aspects of targeted drug delivery [10,49]. The development of computational models can provide predictive information about carrier circulation, interaction with tumor vasculature, tumor accumulation, payload release, efficacy, and safety. In this scenario, they can indicate a very complex system, as well as what factors in the carrier design should be considered to optimize targeted delivery. For instance, a recently published PBPK model [50] was built using data from more than three hundred nanoplatforms as a basis for modeling the delivery of these nanodrugs to the tumor. The final goal of the study was to identify essential factors influencing tumor delivery kinetics using a mechanism-based modeling method as the PBPK approach. Analysis of a model calibrated against this vast amount of experimental data demonstrated that distribution and permeability coefficients at the tumor site, as well as factors associated with them, such as the size and geometry of the nanodrugs, are the key determinants of efficiency of NP delivery to tumors. This finding paves the way for the targeted design of nanodrugs with higher tumor delivery efficiency, while the modeling framework can also be extrapolated to other species (mice were in the original study) to determine the individual optimal dose with minimal side effects.

## 3. Principles of PBPK Modeling of Nanoparticles

**Figure 2:**
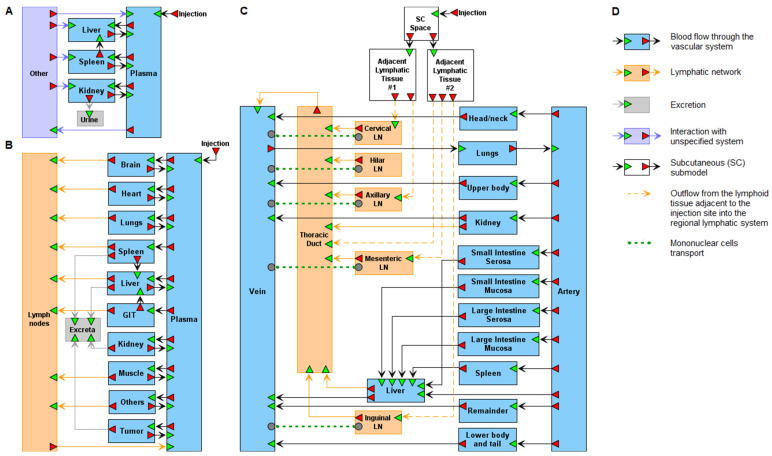
Different in complexity structures of physiologically based pharmacokinetic (PBPK) models of nanoparticle (NP) delivery. (**A**) The model [53] describing the biodistribution of fluorescently labeled NPs in mice used for the controlled delivery of dexamethasone in acute lymphoblastic leukemia therapy. A possible explanation of the “other” compartment (included in the model to fit the simulation results with the experimental data) is the lymphatic system or the adsorption of NPs to the endothelial walls of blood vessels. (**B**) The model of Dogra et al. [54] of whole-body NP pharmacokinetics and their tumor delivery in rats. GIT denotes gastrointestinal tract. (**C**) The model [56] evaluating the systemic and lymphatic pharmacokinetics of 3 HIV drugs, lopinavir, ritonavir, and tenofovir, coformulated in drug-combination NPs (DcNPs). DcNPs administered as a series of subcutaneous (SC) subinjections in the upper back of nonhuman primates are taken up by adjacent lymphoid tissues and then released into the lymphatic system. In regional lymph nodes (LNs), a part of the DcNPs is taken up by mononuclear cells (MCs), while the rest enters the thoracic duct and moves to the bloodstream. Lymphatic recirculation occurs through the organs containing the mononuclear phagocytic system (kidney, spleen, liver). The green dotted lines denote exchange of DcNPs between LN MCs and peripheral blood MCs migrated into the proximity of individual LNs. (**D**) Graphical notation used to visualize the models in the BioUML software, (v. 2022.1, Biosoft.ru, Ltd., Novosibirsk, Russia).

Drug encapsulation into nanocarriers makes it possible to control the pharmacokinetic properties of therapeutics agents, including their release and circulation half-life, while limiting their interactions with healthy tissues [51]. The PBPK modeling approach has existed for many years and has been used to describe time-dependent concentration profiles of substances in various organs of a living system and interspecies scale-up [52]. This approach divides the body into anatomical compartments, interconnected by body fluid systems. The number and nature of the compartments, as well as the complexity of PBPK models, are determined depending on the scientific task and physiological characteristics of the modeled organism. For example, Gilkey et al. considered a five-compartment PBPK model including liver, spleen, kidneys, plasma, and “other” in accordance with the biodistribution of fluorescently labeled NPs in mice used for the controlled delivery of dexamethasone in the therapy of acute lymphoblastic leukemia (Figure 2A) [53]. While to investigate the in silico effects of NP properties, tumor-related variables, and individual physiological differences on systemic bioavailability, mononuclear phagocytic system sequestration, tumor delivery, and excretion of NPs in rats, Dogra et al. developed a tumor-compartment-bearing PBPK model consisting of 12 compartments of interest: brain, heart, lungs, plasma, liver, spleen, gastrointestinal tract, kidneys, muscle, ‘others’, lymph nodes, and a facultative tumor (Figure 2B) [54]. Models designed for primates (including humans) tend to have even more compartments [55,56,57,58]. Thus, Perazzolo et al. investigated a whole-body PBPK model for the anti-HIV drug-loaded NPs in nonhuman primates (Figure 2C) [56]. The model describes the uptake of the injected dose from the subcutaneous site to the adjacent lymphoid depot, passage through the lymph nodes and throughout the lymphatic network, and its subsequent passage into the blood circulation. For this, the model includes 23 compartments: subcutaneous injection site, two adjacent-to-injection lymphoid tissue compartments, thoracic duct, compartments of regional lymph node (cervical node, hilar node, axillary node, mesenteric node, inguinal node), vein, artery, head and neck, lungs, upper body, kidneys, small and large intestines consisting of tissue and mucosa, spleen, liver, lower body and tail, as well as the rest of the body.

**Figure 3:**
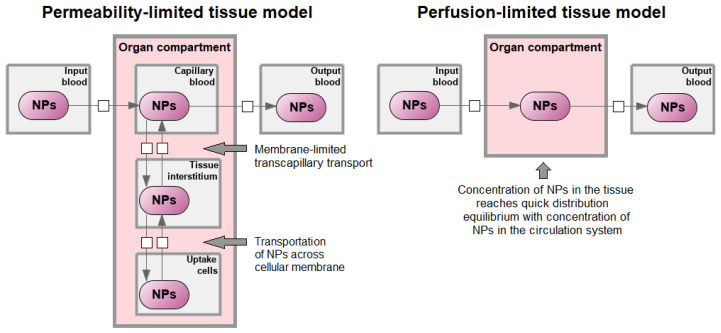
Perfusion-limited and permeability-limited models. The perfusion-limited model assumes that nanoparticles’ (NPs) transportation into tissues is very fast and equilibrium between blood and tissue could be reached instantly. Under such a situation, the transportation of NPs into one tissue depends on its blood supply. In a permeability-limited model, it is assumed that there could be a membrane at the capillary or cellular membrane, or both. Transportation of NPs across cellular membranes is characterized by such mechanisms as endocytic uptake (i.e., phagocytosis, macropinocytosis, and receptor-mediated endocytosis) and exocytic release. Phagocytic cells include reticuloendothelial system cells: monocytes circulating in the blood, Kupffer cells in the liver, reticular cells in the lymph nodes, bone marrow, and spleen, and fixed macrophages of various connective tissues [24,60]. Examples of the perfusion-limited models for NPs can be found in [55,57,60,61]. The permeability-limited models are available in [50,60,61].

Typically, each compartment in PBPK models can be described in two ways (Figure 3). The first is referred to as permeability-limited model (also known as the diffusion-limited or membrane-limited model), in which tissue cell membranes are considered as the main barriers to drug (nanotherapeutic) penetration. The other one is referred to as perfusion-limited model (also known as the flow-limited model) and considers blood perfusion as the only limiting step for drug (nanotherapeutic) penetration through the tissue cell membrane [24,25,59,60]. It is generally accepted that the permeability-limited model is more appropriate for modeling NPs [24,60,61].

It should be noted that the PBPK modeling techniques for NPs and small molecules could be quite different. Traditional route-to-route extrapolation for small molecules is typically performed by using administration of route-specific parameters and keeping other chemical-specific parameters the same [62]. However, unlike small molecules, upon contact with different body fluids following different routes of administration, NPs will be covered by different proteins and other biomolecules, producing different biomolecular coronas and resulting in different patterns of biodistribution. Thus, a recent study by Chou et al. [62] clearly demonstrated that the traditional approach for small molecules is not applicable to NPs, and multiroute PBPK models for NPs should be developed using route-specific data.

The application of mathematical models to nanomedicine is based on breaking down their transport in different discrete and simpler phases which are eventually modeled separately. The sum of these contributions will eventually provide an overall picture of the phenomenon with fundamental hints of prediction that will allow better optimization of cancer treatment and, in this case, NP synthesis [63]. The model developer literally dissects the voyage of the particles in different phases depending on the function of the organ in which they circulate, the barrier function of various elements (i.e., tumor vasculature vs. healthy vasculature), and the characteristics of the carrier (shape, size, surface charge, targeting properties, release rate, etc.). All these phenomena and characteristics are described by mathematical functions that summarize the physical and chemical variabilities that characterize the biological barriers and represent the various parameters under consideration and the core of the modeling. The model is then run with appropriate software, which, by combining different parameters and solving respective equations, provides an estimation of particle location in various organs, including the tumor. The simulated dynamics can eventually be compared with experimental data to optimize the reliability of the calibrated model. When close collaboration between wet-lab biologist and computational scientist is not possible, the latter can extract experimental data from the published literature using “auxiliary” software that allows for the digitization (image-to-number conversion) of images and graphs from the scientific literature and then publish the model under copyright permission (see Section 5). All these transitions are eventually followed up with statistical analysis to evaluate the significance of the data and validate the model. After validation, the model can be used to predict other situations, nanocarriers, and being applied to other tumor diseases. However, an extrapolation of the modeling results from one species to another should be conducted very carefully due to parameter uncertainty. For instance, a collective fitting process of parameter values to data can provide multiple parameter sets that give similar model dynamics reproducing experimental observations. In this case, identifiability of fit parameters is the crucial step of the model analysis for reliable application of PBPK models and a more confident translation of their parameters into new experimental settings [64].

It is worth noting that reproducibility and replicability crisis in research areas has also affected systems biology research such as PBPK modeling in cancer nanomedicine [65]. One attempt to overcome the issue was to develop some of the necessary standards and approaches for building models and represent them by expert researchers in the relevant community. In this context, SBML (Systems Biology Markup Language, an XML-based format) [32] as the most widespread language for defining computational biochemical models and SBGN (Systems Biology Graphic Notation) [33] as a standard for graphical representation of molecular networks have been proposed as gold standards for the representation of biological networks and related models [66]. In addition, these efforts focused on the development of tool-independent ways of presenting models to help avoid potential human errors in translation. Note that these initiatives are not widely ingrained in tools for PBPK modeling. However, some of them described below support these critical standards.

## 4. Main PBPK Modeling Software

**Figure 4:**
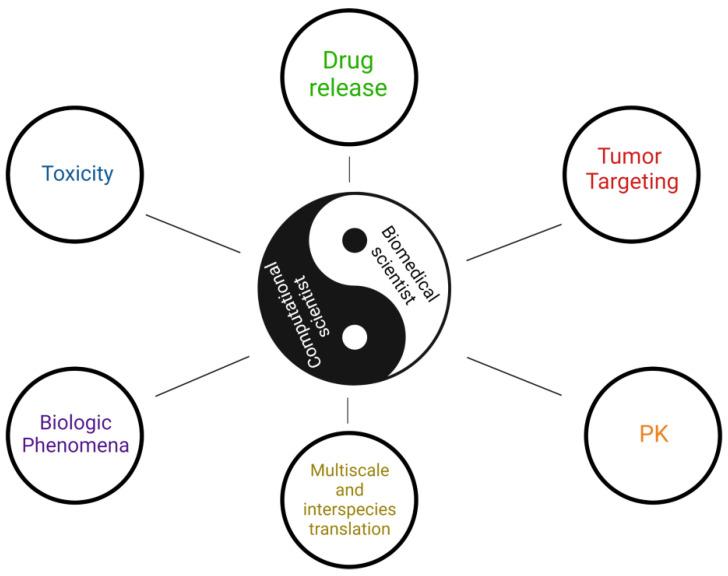
Implementation of biomedical research with computational modeling. Multidisciplinary collaborations between biomedical and computational researchers help to resolve complex issues including nanoparticle toxicity, drug release, targeting, pharmacokinetics, translation into different in vivo models, and understanding biological phenomena.

Below, we provide an overview of the software that are used for PBPK modeling of biodistribution and targeted delivery of NPs. They differ in the language in which they are created and usually specialize in the analysis of specific PBPK situations. They are widely applied and have been used in NP research to predict the potential toxic effect of NPs upon voluntary or accidental administration (i.e., through NP inhalation in polluted environment), determine the PK properties of a payload, facilitate multiscale and interspecies translation, investigate cell biology phenomena, optimize payload encapsulation, and naturally estimate tumor targeting (Figure 4). A comprehensive list of currently available PBPK models for NPs is provided in Supplementary Table S1. A complete list of freely available and commercial software packages used by researchers to develop and analyze these models is given in Supplementary Table S2. We divided these software platforms into simulation software for PBPK modeling (described in this section) and the supporting analysis software compiled in Section 5. Comparative characteristics of simulation platforms are summarized in Table 1.

**MATLAB [67]** is regularly used in many scientific fields, including systems biology [68]. In the case of PBPK models and related PBPK analyses, intermediate to advanced programming skills are required, which present a considerable disadvantage for its widespread use in the field [69]. MATLAB provides a range of mathematical and numerical methods for solving PBPK model equations, parameter estimation, and sensitivity analysis [53,54,55,56,61,70,71,72,73,74,75,76]. In particular, several MATLAB toolkits can be used for modeling, simulating, and analyzing PBPK systems, namely SimBiology [57,77,78,79,80], PottersWheel [81,82], and IntiQuan IQM Tools [70]. MATLAB was used to simulate the biodistribution of NPs of different sizes in the range from 46 to 162 nm after intravenous injection into the plasma compartment of rats [54] (Figure 2B). The authors showed that the model reproduces the in vivo data from a study of the pharmacokinetics of mesoporous silica NPs [83] and then performed local and global sensitivity analyses to rank the importance of various parameters related to the problem of NP delivery to the tumor. Tumor vascularization (fraction and porosity), tumor blood viscosity, NP size and degradation rate have been shown as the main parameters to consider when calculating NP extravasation in the tumor volume, considering passive targeting as the major targeting mechanism. A similar approach was used to evaluate the biodistribution of dexamethasone in a model of acute lymphoblastic leukemia [53] (Figure 2A). The model was based on experimental data obtained after intravenous injection of polymeric NPs. A fluorescent dye was used instead of the drug. The authors noted the difficulties in creating a predictive model, in particular, in the first hours after injections, the model overestimated the blood concentration of the dye. This phenomenon could be explained by particle aggregation and margination [84] in the endothelial wall, and to correct the model, they introduced an additional “other” compartment (i.e., lymphatic system). For longer time points, the model was consistent with the experimental data, demonstrating a rapid accumulation of the particles in the liver and spleen 6 h after their injections. This phenomenon was especially appreciated in consideration that liver and spleen are sites for leukemia blast accumulation and proliferation. MATLAB has also been used to develop a predictive model for tumor targeting of dendrimers functionalized with an insulin-like growth factor 1 peptide analog (NPs for molecular imaging that can bind to mRNA inside cancerous cells) [76]. When fitting the model to the experimental data, the authors found that only 10–20% of the administered NPs were available for transport from the blood to the interstitial tissues and suggested that previous mouse imaging trials used more NPs than necessary. Klapproth et al. [85] used this software to create a model comparing particle biodistribution after intravenous and intratumoral injection. The work focused on superparamagnetic iron oxide NPs for thermal ablation against a potential treatment of brain cancer [86], which is currently being treated with both intravenous and local administration of pharmaceuticals. The model confirmed the experimental data on the dynamic NP redistribution in the organism within 72 h, reaching equilibrium 100 h after local injection. Intravenous injection has demonstrated rapid particle retention in all organs (particularly in the liver and spleen) and subsequent slow release.

**Berkeley Madonna [87]** is relatively easy for beginners and is sufficient to develop basic PBPK models for routine PBPK analyses (e.g., parameter estimation, route-to-route extrapolation, and Monte Carlo analysis) [69,88]. However, it is not intended for visualization of biochemical systems, requiring specialists in the field of biomedicine and mathematical modeling [89]. Examples of PBPK models of NPs coded in Berkeley Madonna are proposed in [50,62,82,90,91,92,93,94,95,96,97,98]. The model by Cheng et al. was used to analyze 376 experimental data sets on the kinetics of NPs in mice with tumors [50]. The authors confirmed that nanomaterials with a hydrodynamic diameter of less than 10 nm can be delivered to tumors with greater efficiency compared to larger particles. In addition, nanomaterials with a hydrodynamic diameter of over 200 nm have a relatively higher efficiency of delivery to the tumor than particles with a size of about 10−200 nm. The study also showed that rod-shaped NPs show better tumor accumulation than variants with other geometry, including spherical, plate-like, or flake-like shapes. Based on several studies, the authors noted that elongated nanostructures, compared to nanospheres, exhibit greater tumor accumulation and a longer half-life in blood circulation, perhaps because of adherence to endothelial cells lining the blood vessel walls, thus, enhancing the site-specific delivery. Furthermore, they concluded that the administration of nanomaterials with a positive (>10 mV) and almost-neutral (−10 to 10 mV) surface charge provides a similar tumor accumulation, which is higher than for negatively charged NPs. These results were partially confirmed by Zhang et al. [97], who used the Berkeley Madonna software to characterize the biodistribution of spherical and rod-shaped gold nanoprobes and tumor accumulation in a model of lung cancer. They found that while nontargeted rod-shaped NPs showed higher tumor accumulation during the first hours after intravenous injection, similar results were obtained at longer time points between nontargeted rod-shaped and spherical delivery platforms. A clear advantage in tumor accumulation was provided by surface functionalization of the particles with RGD to target αvβ3 integrin-positive cancer cells and tumor angiogenic vessels. In the case of active targeting, the authors found a higher distribution coefficient and a much higher maximum tumor uptake rate constant for rod-shaped particles (compared to spherical particles), which eventually resulted in a more effective inhibition of tumor growth by NP-mediated chemoradiotherapy.

**The R language** [99] is a powerful high-level programming platform that is used in various fields of study for statistical computing and graphics [100]. The advantages of the R language are that it is freely distributed and can perform all PBPK analyses. However, it requires medium- to high-level programming skills (same as MATLAB) [69]. The R language may be the optimal choice for projects related to Markov chain Monte Carlo analysis or statistical analysis. For example, Cheng et al. used it for normality testing, one-way ANOVA, and simple and multivariate linear regression to analyze data on the efficiency of NP delivery to tumors [50], whereas Chou et al. applied it to optimize the parameters of a multiroute PBPK model constructed for different sizes (1.4–200 nm) of gold NPs in adult rats at different routes of administration (i.e., intravenous, oral gavage, intratracheal instillation, and endotracheal inhalation) [62]. In addition, the R Shiny package can be used to create an interactive web interface for PBPK models [62].

**acslX**. Many PBPK models for environmental chemicals, drugs, and nanomaterials have been developed using acslX [101], which has been deprecated since November 2015. Lin et al. [69] provided guidance on converting PBPK model code from acslX to alternative modeling tools (Berkeley Madonna, MATLAB, and R) and discussed the advantages and disadvantages of each software package in implementing PBPK models in toxicology. Application of acslX to PBPK modeling of NP delivery can be found in many articles [60,90,91,92,102,103,104,105].

**BioUML** [106] is an integrated web-based platform for systems biology and data analysis [107], which has been successfully tested for modeling biological systems [108,109]. It allows to translate PBPK models from text formats (e.g., Berkeley Madonna) into SBML and SBGN standards, making these models accessible to a wide range of scientists and providing a quick-start guide to work with them [89]. To represent PBPK models in BioUML, a modular approach is used, according to which the system under study is considered as a set of interconnected subsystems (see the example in the Section 6 below). It is also worth noting that BioUML allows one to conduct an identifiability analysis of fitted parameter values mentioned above as a critical procedure for all PBPK models.

**Simcyp Simulator [110]** is a software platform for population PBPK modeling and simulation. It links in vitro data to in vivo absorption, distribution, metabolism, excretion (ADME), and pharmacokinetic/pharmacodynamic outcomes to explore clinical scenarios and support drug development decisions, including regulatory submissions and drug labels [111,112,113,114]. This platform contains a library of predefined models and a database of physiological parameters, making it popular with PBPK users [115,116]. However, due to the complex ADME processes of NPs, it may not provide enough flexibility and capability to support complex NP models [26]. Therefore, the use of Simcyp for the study of nanoformulations is rare [117].

**GastroPlus** [118] is a mechanistically based simulation software package that simulates intravenous, oral, oral cavity, ocular, inhalation, dermal, subcutaneous, and intramuscular absorption, biopharmaceutics, pharmacokinetics, and pharmacodynamics in humans and animals [114,119]. It is widely used for PBPK modeling [116], but, like Simcyp, it is inferior to general purpose programming platforms (Matlab-Simulink, Berkeley Madonna, etc.) in the development of complex PBPK models of NPs [26]. However, GastroPlus has been applied for simulation and in silico prediction of pharmacokinetics and absorption of NPs [120,121,122,123].

**PK-Sim [124]** is a comprehensive software tool for whole-body PBPK modeling. It enables access to all relevant anatomical and physiological parameters for humans and the most common preclinical animal models that are contained in the integrated database [114,125,126]. PK-Sim offers different customized model structures but allows only minor modifications to them. However, it is fully compatible with the stand-alone general purpose graphical modeling tool MoBi [124], which makes the development of PBPK models (also for NPs) possible [125]. Thus, Aborig et al. used the software to parameterize a PBPK model of the biodistribution of gold NPs obtained by green synthesis in mice [70], while Kullenberg et al. applied it to develop a PBPK model describing the disposition of pegylated liposomal doxorubicin [127]. In addition, the platform includes interfaces to R and MATLAB that are useful to analyze and interpret PK-Sim and MoBi models. Advanced model analysis may, for example, involve statistical analysis of the results obtained or the calculation of local or global sensitivity measures to assess model robustness and quality [125].

## 5. Auxiliary PBPK Modeling Software

In this section, we give a brief description of software that is not widely used for PBPK modeling, but can provide additional tools for mathematical analysis:

**Julia** [128] is a flexible dynamic programming language appropriate for scientific and numerical computing. It has been shown that in the context of PBPK models, a DifferentialEquations.jl package outperforms MATLAB in solving a stiff system of ordinary differential equations [72].

**NONMEM** [129] solves pharmaceutical statistical problems in which within-subject and between-subjects variability is taken into account when fitting a PK/PD model to data. Bauer recently created two tutorials to apply this tool to simple and complex scenarios [130,131]. NONMEM allows to build population physiologically based PK models [74].

**GNU MCSim** [132] is specifically designed to conduct Monte Carlo stochastic simulations that can be used for population variability analysis when the distribution of each parameter of the PBPK model is known [69,133]. In particular, the software was applied to estimate parameters in several PBPK models of NPs [58,105,134].

**Phoenix WinNonlin** [135] is one of the most common applications used in the industry for the analysis of PK/PD data. The platform is suitable for modeling, simulations, and analysis of PBPK models of NPs as well [71,136].

**GraphPad Prism** [137] is a commercial scientific software for 2D plotting and statistical analysis [138] which can be applied to evaluate the overall quality of fit between simulated and experimental data [102,103] or to compare delivery efficiency of different types of NPs [50].

**Crystal Ball** [139] is the spreadsheet-based application for predictive modeling, forecasting, simulation, and optimization which is suitable for statistical analysis (e.g., Monte Carlo simulations [74,98] or regression analysis [95]) to parametrize and validate PBPK models.

**TableCurve 2D** [140] is an automated curve fitting software for engineers and researchers that includes a wide range of linear and nonlinear models [141]. It can be used to fit the experimental data on the biodistribution of NP in PBPK simulations [98].

**ADAPT** [142] is a computational modeling platform developed for PK/PD applications. Mager et al. used it to estimate the unknown parameters of the PBPK model for composite nanodevices [82].

**NanoSolveIT** [143] is a new informatics system for in silico nanosafety assessment [144] that, in particular, can be applied for PBPK modeling of nanomaterial biodistribution [145].

**SPSS** [146] is a platform for advanced statistical analysis. Zazo et al. used it in the development of a PBPK model of a drug delivery system based on gold NPs for stavudine biodistribution [136].

**Microsoft Excel** [147] is not a specialized tool for creating PBPK models; however, it can successfully code and analyze them [148,149]. Statistical analysis and calculations with this software can also be used in modeling NP kinetics [105,127].

**Statistics Calculator** [150] was developed by StatPac, Inc. Despite the modest design, the program works correctly and can be used by researchers for statistical analysis of experimental data when creating PBPK models [151].

**Minitab** [152] is a statistics package for identifying trends in data, finding and predicting patterns, uncovering hidden relationships between variables, and visualizing data. An example of a nanoparticle study in which the software was used for two- and three-way ANOVA of the antitumor effect and cellular uptake ratio of free and liposomal doxorubicin in human cancer cell lines (HepG2, Huh7, SNU449, and MCF7) is given in [127].

**COMSOL Multiphysics** [153] is a software package that provides fully coupled multiphysics and single-physics modeling capabilities. This tool is very useful for numerically solving complex physical equations. Thus, Chen et al. applied it to solve the advection-diffusion equations simulating the concentration of superparamagnetic iron oxide NPs coated by gold and conjugated with polyethylene glycol in the cerebral blood and brain tissue in mice [79].

**OriginPro** [154] is a data analysis and graphing software that includes tools for peak fitting, surface fitting, statistics, and signal processing [155]. It can be utilized for parameter estimation of PBPK models [80].

**WebPlotDigitizer** [156] is an open source and cross-platform (web and desktop) semiautomated tool for extraction of the numerical data from engineering images of data visualizations. It works with a wide variety of charts (*x*-*y*, histograms, polar, ternary, maps, etc.) and can be used to digitize experimental data on the biodistribution of NPs published as graphs [50,60,72,102].

**PlotDigitizer** [157] is an open source Java program that converts information from 2D plots or graphs to standard *x*-*y* values (tabular format). For instance, the program can be used to digitize experimental data from the literature, after which PBPK models can be calibrated using the obtained quantitative values [70].

**UN-SCAN-IT** [158] is a software that automatically converts graph images to their underlying (*x*, *y*) data [159]. It works with most image formats (JPG, TIFF, GIF, BMP, PNG, etc.) and can integrate peak areas, smooth data, take derivatives, rescale graphs, and export (*x*, *y*) data for use in other programs. Lee et al. used the software to obtain the PK data for quantum dots from the published figures [160].

## 6. Modular Representation of PBPK Models in BioUML

There are a number of software packages for visual modular modeling of PBPK (Table 1) already described in other studies [26,69,114,115]. Therefore, here, we only focus on BioUML [107], a general purpose software for systems biology that we have been developing since 2002. We recently successfully applied this platform to visual PBPK modeling and we provide a brief description of this example below [89].

Representation of a biological system as a graphical diagram greatly simplifies the construction of a mathematical model, especially in the case of PBPK models divided into compartments (organs and tissues in the body) connected by the circulatory system. To determine such a structure, it is convenient to use the modular mathematical approach [161,162,163] by converting each compartment into a separate module. Therefore, software that supports modular visual modeling, such as BioUML [107], has a distinct advantage over text-based software packages.

**Figure 5:**
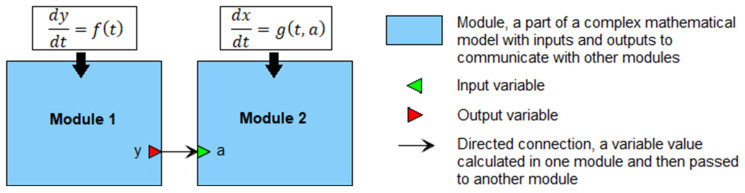
An example of the modular representation of a model that includes two differential equations for *x* and *y*. Module 1 calculates the value of *y* and passes it to Module 2 for initialization of the variable *a*.

A module in BioUML is a part of a complex mathematical model with inputs and outputs to communicate with other modules (Figure 5). The inputs receive variables calculated outside of the module. The outputs pass variables defined inside the module but used outside of it. Each module has its own mathematical base (differential equations, discrete or stochastic systems, etc.) for describing internal processes with varying degrees of detail. In addition, modules can form a hierarchical structure with several levels of nesting.

**Figure 6:**
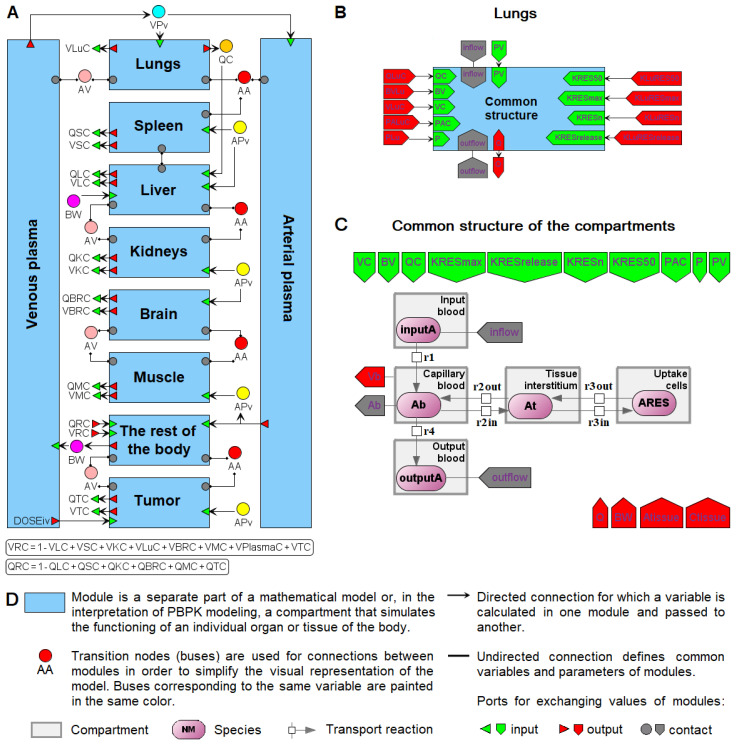
An example of a PBPK model of nanoparticle delivery to solid tumors in mice constructed by Cheng et al. [50] and implemented in BioUML (adapted from [89]). (**A**) Modular representation of the computational model that includes 10 compartments: arterial and venous plasma, lungs, spleen, liver, kidneys, brain, muscle, tumor, and remaining tissues. (**B**) Representation of the lung compartment as a module with common equations of nanoparticle biodistribution in organs that receives specific input values of kinetic parameters. (**C**) Definition of the module of the common compartmental structure, including membrane-limited transcapillary transport, endocytic uptake, and exocytic release of NPs. (**D**) Graphical notation used in BioUML to represent the model in SBML format and SBGN notation.

In the case of PBPK models, different organ compartments are generally designed using the same differential-algebraic equations, but with different values of constants. Therefore, it is logical not only to consider each organ as an individual module, but also to isolate the common equations into a separate submodule that receives specific constants. An example of such a model design is given in [89]. The authors considered an existing PBPK model of nanoparticle delivery to solid tumors in mice [50] and performed the consistent transformation of this model to a hierarchical modular computational form in BioUML (Figure 6). The model consists of 10 compartments, including arterial and venous plasma, as well as organs: lungs, spleen, liver, kidneys, brain, muscles, tumor, and the rest of the body (Figure 6A). Each compartment corresponds to a separate module. Each organ module (excluding the liver) comprises a submodule of common equations for nanoparticle biodistribution (“Common structure” in Figure 6B) and initializes the necessary constant values. The module “Common structure” contains a system of differential-algebraic equations visualized as a chain of reactions of nanoparticle transport between capillary blood, tissue interstitium, and endocytic/phagocytic (or tumor) cells of an organ (Figure 6C). The lungs, spleen, kidneys, muscles, tumor, and the rest of the body include the common equations with nonzero values for all parameters. The brain compartment did not contain an endocytic/phagocytic cell subcompartment in the original model, so the parameters related to it have zero values in “Common structure”. Among the simulated organs, only the liver cannot be expressed through a common system of equations. The difference is that the liver contains Kupffer cells capable of phagocytizing nanoparticles directly from the capillary blood [50]. Therefore, the modules of the liver, venous plasma, and arterial plasma are defined by individual systems of equations. The described modular representation of the PBPK model simplifies its interpretation, editing, and possible development, reducing the risk of technical errors.

## 7. Limitation of the Review

In the current article, we presented an overview of software that can be directly applied to the development of PBPK models for tumor-targeted delivery and biodistribution of NPs. We omitted the description of software platforms that can be used for the general study of NPs, including their toxicity, biocompatibility, biodegradability, or therapeutic and diagnostic properties (e.g., magnetic properties), and other software is worth mentioning for these purposes. For example, processing and visualization of laser scanning microscopy images of cell monolayers exposed to aerosolized NPs can be performed using IMARIS [164], a 3D multichannel image processing software for confocal microscopic images [151]. Particle diameters from transmission electron microscopy images can be analyzed using ImageJ software [97,151,165]. To study the biodistribution of radiolabeled NPs, positron emission tomography (PET) and/or computed tomography is used with an arsenal of software for image reconstruction and processing, such as Tera-Tomo 3D PET, Nucline, and VivoQuant [85]. In addition, a number of other software platforms can be used for quantitative analysis of microscopic images, including CellTracker [166], spatialTIME and iTIME [167], DeepImageJ [168], MCMICRO [169], Viv [170], and others. A more detailed description, comparison, and analysis of these platforms may be the subject of future works.

We considered computational models describing nanoparticle delivery only at the organ level and did not take into account the cellular and molecular levels. However, mathematical models in cancer nanomedicine cover many temporal and spatial orders [41,171]. The modeling of such biological systems requires the use of multiscale approaches that can store information transferred between different scales [48]. Simulation of NP biodistribution is a multistep process that must consider various aspects varying from the production of delivery systems [172,173] to the physical and biochemical processes associated with drug delivery and cellular uptake [174]. Thus, a range of mathematical models considers liposomes as typical delivery systems [175,176,177,178,179]. In this case, the rate of drug release is generally defined as a temperature-dependent function or constant [176,177,178,179]. However, other ways to model the release profile of drugs from nanoliposomes can also be considered. For example, Haghiralsadat et al. investigated the functions of pH and temperature based on the Korsmeyer-Peppas’ model [180,181]:

$$release=k\left(T,pH\right)\cdot t^{n\left(T,pH\right)}$$

Here, *k* depends on the structural characteristics of the liposome and the drug, and *n* is the parameter relative to the drug release mechanism (Fickian diffusion or non-Fickian diffusion) and carrier geometry. At the same time, other models can be investigated to predict the release of a drug from biologically targeted nanocarriers [182,183,184,185,186].

## 8. Conclusions

The solution for many nanomedicine issues might lie in the field of nanoinformatics, which uses computational methods to analyze and process information about the structure and physicochemical characteristics of nanocarriers and nanomaterials [187]. Nanoinformatics has emerged as a new field of research accelerating the understanding of the use, selection, development, and discovery of nanomaterials, as well as NP interactions with biological systems [10,188]. Studying the biodistribution and interaction of engineered NPs with cancer cells, as well as evaluating the effectiveness of treatment using these NPs, covers a wide range of experimental approaches. Modern technologies provide the following experimental techniques: a combination of 2D and 3D optical tomography for in vivo imaging, methods of fluorescent and traditional histology, spectroscopy, flow cytometry, and electron microscopy, methods for obtaining transcriptomic and proteomic data to assess the expression of receptors for various compounds (sugars, lipids, albumin, etc.) [89]. All these techniques provide multiscale and heterogeneous qualitative and quantitative data [189,190,191,192], the integration of which can be based on the PBPK modeling approach [24,25,26,41,52,59]. The application of PBPK models in cancer nanomedicine is still at an early stage to date [41,193]. However, in recent years, a number of models have already been created to study the delivery properties of various types of NPs in tumor-bearing mice [50,76,93,97]. The advantages of such nanomedicine-oriented PBPK models are that they allow studying the distribution of nanodrugs at the site of their action in the body, provide tools for a better understanding of tumor microenvironment complexity, phenotypic diversity, and genetic heterogeneity. In addition, they may prove useful in nanomedicine development to achieve optimal profiles of nanodrug concentrations required in tumor-targeted cells. Finally, they can be used directly for specific cancer patient groups (e.g., the pediatric population) to help guide the development of specific drugs and dosing regimen protocols [193]. To assist in the development of these tools, the scientific community should agree on a minimal amount of data regarding particle characterization, loading and release, and most notably, time and dose used to evaluate the carrier biodistribution that could help the model developers in finding the appropriate data for development of their tools. In this way, we could eventually also accelerate the understanding of the methods used to optimize tumor targeting as well as the biology in terms of mass transport [194] of various tumors.

## Figures and Tables

**Table 1:** Comparison of software packages for PBPK modeling of nanoparticles.

Criteria	MATLAB/ SimBiology	Berkeley Madonna	The R Language	acslX	BioUML	Simcyp Simulator	GastroPlus	PK-Sim/MoBi
Specialized PBPK software	–	–	–	–	–	+	+	+
General purpose software	+	+	+	+	+	–	–	+
Free	–	–	+	+	+	–	–	+
Open source	–	–	+	–	+	–	–	+
Currently supported	+	+	+	–	+	+	+	+
Stand-alone edition	+	+	+	+	+	+	+	+
Web-edition	–	–	–	–	+	+	+	–
Windows	+	+	+	+	+	+	+	+
Linux	+	–	+	–	+	–	–	–
MacOS	+	+	+	–	+	–	+ ^1^	–
Parallel computing	+	–	+	+	+	+	+	+
Requires programming skills	+	+	+	+	–	–	–	–
User-friendly interface	+	+	–	+	+	+	+	+
Interactive web-based interface of a model ^2^	+	–	+	–	+	+	+	–
Visual modeling of the PBPK structure	+	–	–	–	+	–	+	+
Database of models	+	–	–	–	–	+	+	+
Model structural changes	+	+	+	+	+	–	–	+
Monte Carlo simulation	+	+	+	+	–	+	+	+
Parameter estimation	+	+	+	+	+	+	+	+
Sensitivity analysis	+	+	+	+	+	+	+	+
SBML support	+	–	+	+	+	–	–	+
Preferred for NPs ^3^	+	+	+	+	+	–	–	+
Preferred for small molecules ^3^	–	–	–	–	–	+	+	+

^1^ GastroPlus can run on Mac operating systems with Windows virtualization. ^2^ Ability to launch the model through the web (possibly without a web-edition of the software). ^3^ In accordance with the published PBPK models.

## Supplementary Materials

The following supporting information can be downloaded at: https://www.mdpi.com/article/10.3390/ijms232012560/s1. References [195,196,197,198,199] are cited in the supplementary materials.
