# Supplementary material for: Physiologically Based Pharmacokinetic Modeling of Nanoparticle Biodistribution: A Review of Existing Models, Simulation Software, and Data Analysis Tools
Source: Int J Mol Sci. 2022 Oct 19;23(20):12560. doi: 10.3390/ijms232012560 (PMC9604366; doi:10.3390/ijms232012560)
Supplement: Supplementary file 1 [file ijms-23-12560-s001.zip › ijms-1924280-supplementary.pdf]

Table S1 Summary of published physiologically based pharmacokinetic (PBPK) models for nanomaterials (An additional comparison of some of the PBPK models included in the table can also be found in the reviews [1-3]).

Ref	Nanocarriers	Adm. route	Species	Assignment of models	Compartments	Tissue models	Software
Aborig et al., 2019 [4]	16-25 nm coated AuNPs	IP, IV	mouse rat	Biodistribution of AuNPs obtained by green synthesis	Bl, LNs, He, Ki, Mu, Sk, Br, Fat, Go, Li, St, Sp, Pa, SInt, LInt, Bo, Lu	Each organ is divided into Bl, endothelium, macrophages, and interstitial space	IntiQuan IQM Tools, MATLAB Simulations, param. est. PK-Sim Model parametrization PlotDigitizer Image digit.
Bachler et al., 2013 [5]	15-150 nm AgNPs	Derm., oral, Inh.	human rat	Toxic effects of AgNPs	Bl, Br, Sp, Lu, BM, Li, Sk, Te, He, Ki, Mu, Int	Permeability-limited	Not specified
Bachler et al., 2015 [6]	15-150 nm TiO_2 NPs	IV, oral, derm.	mouse rat human	Toxic effects of TiO_2 NPs	Bl, Br, Sk, Lu, St, He, Int, Bo, Li, Sp, Ki, Rem	Permeability-limited	Not specified
Bachler et al., 2015 [7]	2-80 nm AuNPs	ALI	human mouse	Biodistribution of inhaled NPs	Bl, Br, Lu, He, Li, Sp, Ki, Rem	Permeability-limited	Statistics Calculator Stat. an. ImageJ Image an. IMARIS Image an.
Carlander et al., 2016 [8]	13-63 nm naked and PEG PAM NPs, AuNRs, TiO_2 NPs.	IV	rat	Potential toxicity of NPs (their uptake, biodistribution, and elimination)	VBl, ABl, Lu, BM, Br, He, Ki, Li, Sp, Rem	Permeability-limited	acslX Libero Sens. an., param. est., goodness of fit Berkeley Madonna Sens. an.
Carlander et al., 2018 [9]	5-30 nm coated CeO_2 NPs	IV, Inh., IT, oral	rat	Toxicity prediction/biokinetics of CeO_2 NPs	VBl, ABl, Li, Sp, Lu, Ki, He, Br, BM, Rem	Perfusion-limited and permeability-limited	acslX Libero Simulations, param. est. Berkeley Madonna Simulations GraphPad Prism Goodness of fit WebPlotDigitizer Image digit.
Chen et al., 2015 [10]	10-71 nm radioactive ZnO NPs (^{65}ZnO)	IV	mouse	Dynamic interactions of ZnO NPs	Bl, Lu, He, Sp, Li, GIT, Br, Ki, Mu/Bo	Perfusion-limited	Berkeley Madonna Simulations Crystal Ball Param. est. TableCurve 2D Param. est.
Chen et al., 2022 [11]	38-51 nm PEG SPIONs coated by Au	IP	mouse	Impact of an external static magnetic field on the dynamics of magnetic NPs *in vivo* towards the crossing of the blood-brain barrier	VBl, ABl, Li, Sp, Ki, Lu, Br, Rem	Permeability-limited	SimBiology, MATLAB Simulations COMSOL Multiphysics Solution of advection-diffusion equations
Cheng et al., 2020 [12]	Inorganic and organic NPs	IV	mouse	Tumor delivery efficiency of nanomedicines	VBl, ABl, Lu, Sp, Li, Ki, Br, Mu, Tu, Rem	Permeability-limited	Berkeley Madonna Param. est. R language Stat. an. GraphPad Prism Stat. an. WebPlotDigitizer Image digit.
Chou et al., 2022 [13]	1.4-200 nm AuNPs	IV, oral gav., IT, Inh.	rat	Testing that the route-to-route extrapolation approach used for small molecules is not appropriate for NPs	VBl, ABl, UAir, Trach, Sp, Li, GIT, Ki, Rem	Permeability-limited	Berkeley Madonna Simulations, param. est. R language Markov chain Monte Carlo simulation, param. est., stat. an., web interactivity (using R Shiny)

Deng et al., 2019 [14]	13-100 nm PEG AuNPs	IV	mouse<br>human	Biodistribution of PEG AuNPs	VBl, ABl, Lu, Br, Sp, Li, Ki, Rem	Permeability-limited	acslX Simulations, param. est.
Dogra et al., 2020 [15]	46-162 nm PEG trimethylsilane	IV	rat	The effects of NP properties, tumor variables, and individual physiological differences on the systemic bioavailability, MPS sequestration, tumor delivery, and excretion of NPs	Bl, Br, He, Lu, Li, Sp, GIT, Ki, Mu, LNs, Tu, Rem	Br, He, Lu, GIT, Ki, Mu, Tu, and Rem consist of vascular and extravascular parts. Li and Sp contain macrophages	MATLAB Simulations, param. est., sens. an.
Dong et al., 2015 [16]	203 nm nanocrystals of SNX-2112	IV	rat	Disposition of SNX-2112 (Hsp90 inhibitor for cancer treatment) after Adm. of the drug nanocrystals	Bl, Lu, He, Ki, Sp, Li, Int, Rem	Perfusion-limited	MATLAB Simulations<br>Phoenix WinNonlin Compartmental/non-compartmental analysis of Bl/tissue concentrations vs. time
Dubaj et al., 2022 [17]	13 nm PEG AuNPs	CT, IV	human<br>rat	Comparison of PEG AuNP biodistribution in rats with that predicted by a PBPK model calibrated on the internalized amount of Au in individual human cell lines	Bl, Li, Sp, Lu, Ki	Perfusion-limited	SimBiology, MATLAB Simulations<br>OriginPro Param. est.
Elgrabli et al., 2015 [18]	<25 nm TiO_2 NPs	IV	rat	Time course of TiO_2 concentrations in organs	VBl, ABl, Lu, Sp, Li	Perfusion-limited	GNU MCSim Param. est., stat. an.
Gilkey et al., 2015 [19]	Polymeric NPs	IV	mouse	Disposition of NPs for the delivery of DEX in acute lymphoblastic leukemia therapy	Bl, Li, Sp, Ki, Rem	Perfusion-limited	MATLAB Quantitative analysis
Glass et al., 2022 [20]	4-100 nm PEG AuNPs	IV	mouse	Continuous biodistribution of NPs	Model A: Lu, He, Ki, Li, Sp;<br>Model B: Lu, He, Ki, Li, Sp, Int, Rem	Each compartment consists of: vascular space, endothelial cell, tissue	MATLAB Simulations<br>Julia Simulations<br>WebPlotDigitizer Image digit.
Howell, Chauhan, 2010 [21]	PEG anionic liposomes	IV	human	Design of liposome therapy for treatment of overdoses of tricyclic antidepressants and local anesthetics	VBl, ABl, Lu, Fat, Br, He, Li, Pa, Int, Sp, Thym, Bo, Sk, Mu, Ki	Perfusion-limited	MATLAB Simulations
Kagan et al., 2014 [22]	<100 nm liposomes	IV	mouse<br>rat<br>human	Disposition of AmB, an agent for the treatment of severe fungal or parasitic infections, after liposomal Adm.	Bl, Lu, He, Ki, GIT, Li, Sp, Rem	Each tissue compartment is divided into vascular and extravascular parts	MATLAB Simulations
Kasyanova, Bazhukova, 2020 [23]	CeO_2 NPs	IV	rat	Potential toxicity of CeO_2 NPs; their absorption, distribution, metabolism, and excretion in the body	VBl, ABl, Lu, Br, He, Li, Sp, GIT, Ki, Rem	Perfusion-limited and permeability-limited	SimBiology, MATLAB Simulations, param. est., goodness of fit
Klapproth et al., 2020 [24]	60-130 nm SPIONs	Intratum., IV	mouse	Biodistribution of SPIONs in a GL261 xenograft glioblastoma model	Bl, Lu, He, Ki, Sp, Bo, Sk, St, Li, Int, LInt, Br, Mu, Tu	The change of NP amount in each compartment is a function of time	MATLAB Simulations, param. est<br>Tera-Tomo 3D PET image an.<br>Nucline Image an.<br>VivoQuant Image an.

Kullenberg et al., 2021 [25]	40 nm PEG liposomes	CT	human	Simulation of drug concentration-time curves for PEG liposomal DOX and free DOX in primary liver cancer cell lines (HepG2, Huh7, SNU449) and breast cancer cell line (MCF7)	Bl, Li, Ki, Sp, Lu, Fat, Mu, Bo, He, Sk, Int, Pa	PEG liposomal DOX was modeled as an entity limited to vascular and interstitial distribution	PK-Sim Simulations<br>Minitab Stat. an.<br>Excel Calculations
Lankveld et al., 2010 [26]	20-110 nm AgNPs	IV	rat	Kinetics and toxicity of AgNPs	Bl, Li, Sp, Ki, Rem	Li, Sp, Ki contain NPs that are exchanged between tissue and Bl, and NPs (quasi) irreversibly incorporated into tissue	ACSL Simulations, param. est.
Lee et al., 2009 [27]	12-80 nm QDs	IV, SC	mouse<br>rat	Biodistribution of QDs	Bl, Sk, Mu, Ki, Li, Rem	Perfusion-limited	acslXtreme Simulations, param. est.<br>UN-SCAN-IT Image digit.
Li et al., 2012 [28]	40-150 nm PLGA-MPEG NPs	IV	mouse	Biodistribution of PLGA-MPEG NPs	VBl, ABl, Lu, GIT, GI lumen, Li, Sp, Ki, Ur, Rem	Perfusion-limited or permeability-limited	MATLAB Simulations, param. est.
Li et al., 2014 [29]	35 nm PEG PAM NPs	IV	rat	Biodistribution of PEG PAM NPs	VBl, ABl, Lu, BM, Br, He, Ki, Li, Sp, Rem	Perfusion-limited and permeability-limited	Berkeley Madonna Simulations<br>acslX Simulations, param. est.
Li et al., 2015 [30]	25-90 nm new and aged CeO_2 NPs	Inh.	rat	Toxic effects and biodistribution kinetic of the inhaled CeO_2 NPs	VBl, ABl, UAir, Lu, GIT, Li, Sp, Ki, He, Br, Rem	Perfusion-limited and permeability-limited	Berkeley Madonna Simulations<br>acslX Simulations, param. est.
Li et al., 2021 [31]	80-200 nm polymeric NPs	IV	mouse	Dynamics of NPs within and between organs in tumor-bearing mice	VBl, ABl, He, Li, Sp, Lu, Ki, Tu, Rem	Perfusion-limited or permeability-limited	Berkeley Madonna Simulations, param. est.
Liang et al., 2016 [32]	3.5-4.2 nm Cd-containing QDs	IV, SC	mouse	Accurately characterizing and predicting the *in vivo* fate of long-circulating inorganic NPs	VBl, ABl, Lu, Sp, Li, Ki, Rem	Permeability-limited	Berkeley Madonna Simulations, param. est.
Lin et al., 2008 [33]	18.5 nm Qtracker 705 QDs	IV	mouse	Prediction of the time-dependent kinetic and distributional changes of QD705 in tissues	VBl, ABl, Ki, Li, Sp, Rem	The time-dependent tissue distribution coefficients are given by Hill equations	Berkeley Madonna Simulations<br>Crystal Ball Regression analysis to obtain coefficients for Hill equations
Lin et al., 2016 [34]	13-100 nm PEG AuNPs	IV	mouse	Understanding the PK of PEG-coated AuNPs	VBl, ABl, Lu, Br, Sp, Li, Ki, Rem	Perfusion-limited or permeability-limited	acslX Simulations, param. est.<br>WebPlotDigitizer Image digit.
Lin et al., 2016 [35]	13-100 nm coated AuNPs	IV	mouse rat<br>pig<br>human	Tissue distribution and toxicity assessment of AuNPs	VBl, ABl, Lu, Br, Sp, Li, Ki, Rem	Permeability-limited	acslX Simulations<br>GraphPad Prism Goodness of fit
Lu et al., 2016 [36]	111.6 nm coated liposomes	IV	rat<br>mouse<br>human	Disposition of docetaxel, semisynthetic taxoid prescribed for several kinds of malignancies, after Adm. of modified liposomes	VBl, ABl, Lu, Br, He, Sp, Li, Int, Ki, Mu, Rem	Perfusion-limited or permeability-limited with or without deep tissue subcompartment	NONMEM Population modeling<br>MATLAB Graphs, stat. an.<br>Crystal Ball Monte Carlo simulation

MacCalman et al., 2009 [37]	15-20 nm IrNPs and AgNPs	IT, Inh.	rat	Translocation of inhaled NPs from lungs to different target organs	OR, UAir, AR, In, LNs, VBl, ABl, GIT, Li, Ki, He, Sp, Br, Rem	Each organ includes subcompartments for the tissue, capillary and sequestration	MATLAB Param. est.
Mager et al., 2012 [38]	5-22 nm Au/dendrimer NPs	IV	mouse	Temporal exposure and elimination of gold/dendrimer CNDs	Bl, Lu, He, Ki, Ur, Mu, Br, Sp, Li, Fe, Rem	Permeability-limited	Berkeley Madonna Simulations PottersWheel, MATLAB Sens. an. ADAPT Param. est.
Opitz et al., 2010 [39]	Dendrimers	IV	mouse	PK of molecular imaging NPs for mRNA detection determined in tumor-bearing mice	VBl, ABl, Lu, Mu, He, Sp, SInt/St, Li, LInt, Ki, Tu, Rem	Permeability-limited	MATLAB Simulations
Perazzolo et al., 2022 [40]	52 nm lipid NPs	SC	nonhuman primate	Systemic and lymphatic PK of three HIV drugs (lopinavir, ritonavir and tenofovir) contained in drug-combination NPs	SC site, 2 ALTs, TD, 5 LNs (cervical, hilar, axillary, mesenteric, inguinal), VBl, ABl, H&N, Lu, UBody, Ki, SInt serosa, SInt mucosa, LInt serosa, LInt mucosa, Sp, Li, LBody, Rem	Perfusion-limited and permeability-limited	MATLAB Simulations, param. est.
Péry et al., 2009 [41]	5-10 nm (99m)Tc-labeled CNPs	Inh.	human	Analysis of imaging data to describe the absorption and distribution of Tc-labelled CNPs	VBl, ABl, Air, Lu, Br, He, BM, Ad, Thyr, Ki, Ur, Fat, Mu, Sk, Bre, Pa, Sp, St, St lumen, Int, Gut lumen, Li, Te, Rem	Perfusion-limited	GNU MCSim Numerical integration, param. est., Markov chain Monte Carlo simulation
Rajoli et al., 2015 [42]	Solid NPs	IM	human	Predicting PK of antiretrovirals (tenofovir, emtricitabine, efavirenz, rilpivirine, etravirine, raltegravir, dolutegravir, atazanavir) after Adm. of solid drug NPs	VBl, Abl, Fat, Bo, Br, Mu, Sk, Go, Thym, Lu, He (including LV and RV), IM depot, CBl, Pa, Sp, St tissue (including St), PV, SInt (7 parts), Li, Ki	Perfusion-limited	SimBiology, MATLAB Simulations
Silva et al., 2017 [43]	21 nm coated SPIONs	IV	mouse	Predicting PK of SPIONs	Bl, He, Sp, Te, Br, Mu, Fat, Int, Ki, Lu, Li, Sk	Passive diffusion via capillary endothelium; active uptake in Li, Sp, Lu by macrophages	SimBiology, MATLAB Simulations
Sweeney et al., 2015 [44]	15-20 nm IrNPs (^{192}Ir)	IT	rat	Long-term kinetics of metal NPs	OR, UAir, AR, In, LNs, VBl, ABl, GIT, Gut contents, Li, Ki, He, Sp, Br, Fe, Ur, Rem	Perfusion-limited	GNU MCSim Simulations, param. est. acslX Sens. an., Monte Carlo an. Excel Graphs, stat an.
Tsiros et al., 2022 [45]	20-22 nm TiO_2 NPs	Inh.	rat human	Whole-body biodistribution and long-term kinetics of NMs	VBl, ABl, UAir, Lu, Li, Sp, Ki, He, Br, Ut, Sk, Rem	Permeability-limited	NanoSolveIT Simulations

Wenger et al., 2011 [46]	31 nm naked and PEG PAM NPs	IV	rat	Distribution dynamics of naked and PEG PAM NPs	Bl, tissues with slow deposition, tissues with fast deposition, Ur, Fe	Rapidly/slowly perfused tissues form two distinct compartments	Berkeley Madonna Simulations
Zazo et al., 2022 [47]	40 nm AuNPs	IP	rat	Biodistribution of stavudine, an antiretroviral drug, after its Adm. in AuNPs	Bl, Br, Li, Sp, Thym, Rem	Permeability-limited	Phoenix WinNonlin Simulations, non-compartmental analysis<br>SPSS Stat. an.<br>GraphPad Prism Stat. an.
Zhang et al., 2019 [48]	56 nm $\alpha v\beta 3$ targeted spherical and rod- shaped AuNPs	IV	mouse	Revealing the mechanism of NPs' shape and active targeting ligands effects on tumor accumulation	VBl, ABl, Lu, Sp, Li, Ki, Tu, Rem	Permeability-limited	Berkeley Madonna Simulations, param. est.<br>ImageJ Image digit.

Abbreviations: ABl=Arterial blood; Ad=Adrenals; Adm.=Administration; AgNPs = Silver NPs; Air=Airways; ALI= Air-liquid interface; ALT=Adjacent-to-injection lymphoid tissue; AmB =Amphotericin B; An. =Analysis; AR=Alveolar region; Au = Gold; AuNPs = Gold NPs; AuNRs = Gold NRs; Bl=Blood; BM=Bone marrow; Bo=Bones; Br=Brain; Bre=Breast; CBl=Capillary blood; Cd = Cadmium; CeO_2 = Cerium dioxide; CNPs = carbon NPs; CT=Cell treatment; Derm.=Dermal; Fe=Feces; DEX=dexamethasone; digit.=Digitalization; DOX=Doxorubicin; gav.=Gavage; GI=Gastrointestinal; GIT=Gastrointestinal tract; Go=Gonads; H&N=Head and neck; He=Heart; IM=Intramuscular; In=Interstitium; Inh.=Inhalation; Int=Intestine; Intratum=Intratumural; IP=Intraperitoneal; IrNPs = Iridium NPs; IT= intratracheal; IV=Intravenous; Ki=Kidneys; LBody=Lower body and tail; Li=Liver; L Int=Large intestine; LN=Lymph node; Lu=Lungs; LV=Left ventricle; MPEG = Monomethoxypoly (ethylene glycol); MPS= Mononuclear phagocytic system; Mu= Muscle; NPs=Nanoparticles; NRs = Nanorods; OR=Olfactory region; Pa=Pancreas; PAM = Polyacrylamide; Param. Est.=Parameter estimation; PEG = Pegylated; PK=Pharmacokinetics; PLGA = Poly(lactic-co-glycolic) acid; PV=Portal vein; QDs = Quantum dots; Rem=Remainder; RV=Right ventricle; SC=Subcutaneous; Sens.=Sensitivity; SInt=Small intestine; Sk=Skin; Sp=Spleen; SPIONs=Superparamagnetic Iron Oxide nanoparticles; St=Stomach; Stat.=Statistical; Tc = Technetium; TD=Thoracic duct; Te=Testes; Thym=Thymus; Thyr=Thyroid; Trach=Tracheobronchial; TiO_2 = Titanium dioxide; Tu=Tumor; UAir=Upper airways; UBody=Upper body; Ur=Urine; Ut=Uterus; VBl=Venous blood; ZnO=zinc oxide

Table S2. Software used for development of PBPK models of nanoparticles with examples listed in Table S1.

Software/trademarks	Developers	Primary use	Access
ACSL, acslX, acslX Libero, acslXtreme [49]	Aegis Technologies Group, Inc., USA	General-purpose software for modeling	Not supported since November 2015
ADAPT<br>https://bmsr.usc.edu/software/adapt/	Biomedical Simulations Resource (BMSR), USA	Specialized software for PK/PD modeling	No charge to the user, under the terms of a Release Agreement
Berkeley Madonna [50]<br>https://berkeley-madonna.myshopify.com/	University of California at Berkeley, USA	General-purpose software for modeling	A free version with slightly limited functionality; a licensed version that is registered to individuals
BioUML [51]<br>https://www.biouml.org	Biosoft.ru, Ltd., Russia	General-purpose software for modeling	Free and open source stand-alone and web versions. Source files are available in BioStore: https://bio-store.org/
COMSOL Multiphysics<br>https://www.comsol.com/	COMSOL Inc., USA and other countries	General-purpose software for physics and engineering applications	Commercial
Crystal Ball<br>https://www.oracle.com/applications/crystalball/	Decisioneering, Inc., USA (until March 2007);<br>Hyperion, USA (until July 2007);<br>Oracle, USA	Data analysis software	Commercial
GastroPlus [52]<br>https://www.simulations-plus.com/software/gastroplus/	Simulations Plus, Inc., USA	Specialized PBPK software	Commercial
GNU MCSim [53]<br>https://www.gnu.org/software/mcsim	GNU Project, Free Software Foundation, Inc., USA	General-purpose software for modeling	Free
GraphPad Prism [54]<br>https://www.graphpad.com/	GraphPad Software, Inc., USA	Data analysis software; graphical representation of data	Commercial
DifferentialEquations.jl, the Julia package [55]	Chris Rackauckas (lead developer), et al., USA	A package of differential equation solvers and machine learning components	Free and open source:<br>https://github.com/SciML/DifferentialEquations.jl
MATLAB [56]<br>http://www.mathworks.com	The MathWorks, Inc., USA	General-purpose software for modeling	Commercial
Microsoft Excel [57]<br>https://www.microsoft.com/en-us/microsoft-365/excel	Microsoft, USA	Data analysis software; graphical representation of data	Commercial
Minitab<br>https://www.minitab.com/en-us/	Minitab, LLC, USA	Data analysis software; graphical representation of data	Commercial with a free 30-day trial
NanoSolveIT [58]<br>https://nanosolveit.eu/	Consortium of 16 European partners and 8 international partners	Specialized software for testing and risk assessment of nanomaterials for humans and the environment	Available both as stand-alone open source software and through a cloud platform:<br>https://github.com/NanoSolveIT

NONMEM [59,60]<br>https://www.iconplc.com/innovation/nonmem	Beal, S.L., Sheiner, L.B. (University of California, USA), Bauer, R.J. (ICON Clinical Research, LLC, Ireland)	Specialized software for PK/PD modeling	Commercial
OriginPro [61]<br>https://www.originlab.com/origin	OriginLab Corporation, USA	Data analysis software; graphical representation of data	Commercial
Phoenix WinNonlin<br>https://www.certara.com/software/phoenix-winnonlin/	Certara, LP, USA	Specialized software for PK/PD modeling	Commercial
PK-sim/MoBi [62,63]<br>https://www.open-systems-pharmacology.org/	Bayer Technology Services, GmbH, Germany	Specialized PBPK software; general-purpose software for modeling	Free and open source:<br>https://github.com/Open-Systems-Pharmacology/PK-Sim/<br>https://github.com/Open-Systems-Pharmacology/MoBi
PlotDigitizer<br>https://plot-digitizer.software.informer.com/	Huwaldt, J.A.	Image digitalization software	Free and open source:<br>https://sourceforge.net/projects/plotdigitizer/files/plotdigitizer/
R language [64]<br>https://www.r-project.org/	R Core Team	General-purpose software for modeling; data analysis software	Free and open source:<br>https://github.com/wch/r-source
Simcyp Simulator [65,66]<br>https://www.certara.com/software/simcyp-pbpk/	Certara, LP, USA	Specialized PBPK software	Commercial
SPSS<br>https://www.ibm.com/products/spss-statistics	SPSS, Inc., USA (until 2009);<br>IBM, USA	Data analysis software	Commercial
Statistics Calculator<br>https://statistics-calculator.software.informer.com/	StatPac, Inc., USA	Data analysis software	Free
TableCurve 2D [67]<br>https://systatsoftware.com/tablecurve2d/	AISN Software, Inc. (until 1990);<br>Jandel Scientific Software, USA (until 1995);<br>SPSS, Inc., USA (until 2004);<br>Systat Software, Inc., USA	Data analysis software	Commercial
UN-SCAN-IT [68]<br>https://www.silkscientific.com/graph-digitizer.htm	Silk Scientific, Inc., USA	Image digitalization software	Commercial
WebPlotDigitizer<br>https://automeris.io/WebPlotDigitizer/	Rohatgi, A.	Image digitalization software	Free and open source:<br>https://github.com/ankitrohatgi/WebPlotDigitizer

References

1. Lin, Z.; Monteiro-Riviere, N.A.; Riviere, J.E. Pharmacokinetics of metallic nanoparticles. *Wiley Interdisciplinary Reviews: Nanomedicine and Nanobiotechnology* **2015**, *7*, 189-217.
2. Utembe, W.; Clewell, H.; Sanabria, N.; Doganis, P.; Gulumian, M. Current approaches and techniques in physiologically based pharmacokinetic (PBPK) modelling of nanomaterials. *Nanomaterials* **2020**, *10*, 1267.
3. Li, M.; Zou, P.; Tyner, K.; Lee, S. Physiologically based pharmacokinetic (PBPK) modeling of pharmaceutical nanoparticles. *The AAPS journal* **2017**, *19*, 26-42.
4. Aborig, M.; Malik, P.R.; Nambiar, S.; Chelle, P.; Darko, J.; Mutsaers, A.; Edginton, A.N.; Fleck, A.; Osei, E.; Wettig, S. Biodistribution and physiologically-based pharmacokinetic modeling of gold nanoparticles in mice with interspecies extrapolation. *Pharmaceutics* **2019**, *11*, 179.
5. Bachler, G.; von Goetz, N.; Hungerbühler, K. A physiologically based pharmacokinetic model for ionic silver and silver nanoparticles. *International journal of nanomedicine* **2013**, *8*, 3365.
6. Bachler, G.; von Goetz, N.; Hungerbuhler, K. Using physiologically based pharmacokinetic (PBPK) modeling for dietary risk assessment of titanium dioxide (TiO2) nanoparticles. *Nanotoxicology* **2015**, *9*, 373-380.
7. Bachler, G.; Losert, S.; Umehara, Y.; von Goetz, N.; Rodriguez-Lorenzo, L.; Petri-Fink, A.; Rothen-Rutishauser, B.; Hungerbuehler, K. Translocation of gold nanoparticles across the lung epithelial tissue barrier: Combining in vitro and in silico methods to substitute in vivo experiments. *Particle and fibre toxicology* **2015**, *12*, 1-18.
8. Carlander, U.; Li, D.; Jolliet, O.; Emond, C.; Johanson, G. Toward a general physiologically-based pharmacokinetic model for intravenously injected nanoparticles. *International journal of nanomedicine* **2016**, *11*, 625.
9. Carlander, U.; Moto, T.P.; Desalegn, A.A.; Yokel, R.A.; Johanson, G. Physiologically based pharmacokinetic modeling of nanoceria systemic distribution in rats suggests dose-and route-dependent biokinetics. *International Journal of Nanomedicine* **2018**, *13*, 2631.
10. Chen, W.-Y.; Cheng, Y.-H.; Hsieh, N.-H.; Wu, B.-C.; Chou, W.-C.; Ho, C.-C.; Chen, J.-K.; Liao, C.-M.; Lin, P. Physiologically based pharmacokinetic modeling of zinc oxide nanoparticles and zinc nitrate in mice. International journal of nanomedicine **2015**, 10, 6277.
11. Chen, J.; Yuan, M.; Madison, C.A.; Eitan, S.; Wang, Y. Blood-brain barrier crossing using magnetic stimulated nanoparticles. Journal of Controlled Release **2022**, 345, 557-571.
12. Cheng, Y.-H.; He, C.; Riviere, J.E.; Monteiro-Riviere, N.A.; Lin, Z. Meta-analysis of nanoparticle delivery to tumors using a physiologically based pharmacokinetic modeling and simulation approach. *ACS nano* **2020**, *14*, 3075-3095.
13. Chou, W.C.; Cheng, Y.H.; Riviere, J.E.; Monteiro-Riviere, N.A.; Kreyling, W.G.; Lin, Z. Development of a multi-route physiologically based pharmacokinetic (PBPK) model for nanomaterials: a comparison between a traditional versus a new route-specific approach using gold nanoparticles in rats. *Particle and fibre toxicology* **2022**, *19(1)*, 47.
14. Deng, L.; Liu, H.; Ma, Y.; Miao, Y.; Fu, X.; Deng, Q. Endocytosis mechanism in physiologically-based pharmacokinetic modeling of nanoparticles. *Toxicology and Applied Pharmacology* **2019**, *384*, 114765.
15. Dogra, P.; Butner, J.D.; Ramírez, J.R.; Chuang, Y.-l.; Noureddine, A.; Brinker, C.J.; Cristini, V.; Wang, Z. A mathematical model to predict nanomedicine pharmacokinetics and tumor delivery. *Computational and structural biotechnology journal* **2020**, *18*, 518-531.
16. Dong, D.; Wang, X.; Wang, H.; Zhang, X.; Wang, Y.; Wu, B. Elucidating the in vivo fate of nanocrystals using a physiologically based pharmacokinetic model: a case study with the anticancer agent SNX-2112. *International Journal of Nanomedicine* **2015**, *10*, 2521.
17. Dubaj, T.; Kozics, K.; Sramkova, M.; Manova, A.; Bastús, N.G.; Moriones, O.H.; Kohl, Y.; Dusinska, M.; Runden-Pran, E.; Puntes, V.; Nelson, A.; Gabelova, A.; Simon, P. Pharmacokinetics of PEGylated gold nanoparticles: in vitro-in vivo correlation. *Nanomaterials (Basel)* **2022**, *12(3)*, 511.
18. Elgrabli, D.; Beaudouin, R.; Jbilou, N.; Floriani, M.; Pery, A.; Rogerieux, F.; Lacroix, G. Biodistribution and clearance of TiO2 nanoparticles in rats after intravenous injection. *PLoS One* **2015**, *10*, e0124490.

19. Gilkey, M.; Krishnan, V.; Scheetz, L.; Jia, X.; Rajasekaran, A.; Dhurjati, P. Physiologically based pharmacokinetic modeling of fluorescently labeled block copolymer nanoparticles for controlled drug delivery in leukemia therapy. *CPT: pharmacometrics & systems pharmacology* **2015**, *4*, 167-174.
20. Glass, E.; Kulkarni, S.; Eng, C.; Feng, S.; Malavia, A.; Radhakrishnan, R. Physiologically Based Multiphysics Pharmacokinetic Model for Determining the Temporal Biodistribution of Targeted Nanoparticles, **2022**.
21. Howell, B.A.; Chauhan, A. A physiologically based pharmacokinetic (PBPK) model for predicting the efficacy of drug overdose treatment with liposomes in man. *Journal of pharmaceutical sciences* **2010**, *99*, 3601-3619.
22. Kagan, L.; Gershkovich, P.; Wasan, K.M.; Mager, D.E. Dual physiologically based pharmacokinetic model of liposomal and nonliposomal amphotericin B disposition. *Pharmaceutical research* **2014**, *31*, 35-45.
23. Kasyanova, V.; Bazhukova, I. Modeling of cerium oxide nanoparticles pharmacokinetics. In Proceedings of the AIP Conference Proceedings, **2020**; p. 080015.
24. Klapproth, A.P.; Shevtsov, M.; Stangl, S.; Li, W.B.; Multhoff, G. A new pharmacokinetic model describing the biodistribution of intravenously and intratumorally administered superparamagnetic iron oxide nanoparticles (SPIONs) in a GL261 xenograft glioblastoma model. *International Journal of Nanomedicine* **2020**, *15*, 4677.
25. Kullenberg, F.; Degerstedt, O.; Calitz, C.; Pavlović, N.; Balgoma, D.; Gråsjö, J.; Sjögren, E.; Hedeland, M.; Heindryckx, F.; Lennernäs, H. In vitro cell toxicity and intracellular uptake of doxorubicin exposed as a solution or liposomes: implications for treatment of hepatocellular carcinoma. *Cells* **2021**, *10(7)*, 1717.
26. Lankveld, D.P.; Oomen, A.G.; Krystek, P.; Neigh, A.; Troost–de Jong, A.; Noorlander, C.; Van Eijkeren, J.; Geertsma, R.; De Jong, W. The kinetics of the tissue distribution of silver nanoparticles of different sizes. *Biomaterials* **2010**, *31*, 8350-8361.
27. Lee, H.A.; Leavens, T.L.; Mason, S.E.; Monteiro-Riviere, N.A.; Riviere, J.E. Comparison of quantum dot biodistribution with a blood-flow-limited physiologically based pharmacokinetic model. *Nano letters* **2009**, *9*, 794-799.
28. Li, M.; Panagi, Z.; Avgoustakis, K.; Reineke, J. Physiologically based pharmacokinetic modeling of PLGA nanoparticles with varied mPEG content. *International journal of nanomedicine* **2012**, *7*, 1345.
29. Li, D.; Johanson, G.; Emond, C.; Carlander, U.; Philbert, M.; Jolliet, O. Physiologically based pharmacokinetic modeling of polyethylene glycol-coated polyacrylamide nanoparticles in rats. *Nanotoxicology* **2014**, *8*, 128-137.
30. Li, D.; Morishita, M.; Wagner, J.G.; Fatouraie, M.; Wooldridge, M.; Eagle, W.E.; Barres, J.; Carlander, U.; Emond, C.; Jolliet, O. In vivo biodistribution and physiologically based pharmacokinetic modeling of inhaled fresh and aged cerium oxide nanoparticles in rats. *Particle and fibre toxicology* **2015**, *13*, 1-20.
31. Li, L.; He, H.; Jiang, S.; Qi, J.; Lu, Y.; Ding, N.; Lin, H.-S.; Wu, W.; Xiang, X. Simulation of the in vivo fate of polymeric nanoparticles traced by environment-responsive near-infrared dye: A physiologically based pharmacokinetic modelling approach. *Molecules* **2021**, *26*, 1271.
32. Liang, X.; Wang, H.; Grice, J.E.; Li, L.; Liu, X.; Xu, Z.P.; Roberts, M.S. Physiologically based pharmacokinetic model for long-circulating inorganic nanoparticles. *Nano Letters* **2016**, *16*, 939-945.
33. Lin, P.; Chen, J.-W.; Chang, L.W.; Wu, J.-P.; Redding, L.; Chang, H.; Yeh, T.-K.; Yang, C.S.; Tsai, M.-H.; Wang, H.-J. Computational and ultrastructural toxicology of a nanoparticle, Quantum Dot 705, in mice. *Environmental science & technology* **2008**, *42*, 6264-6270.
34. Lin, Z.; Monteiro-Riviere, N.A.; Riviere, J.E. A physiologically based pharmacokinetic model for polyethylene glycol-coated gold nanoparticles of different sizes in adult mice. *Nanotoxicology* **2016**, *10*, 162-172.
35. Lin, Z.; Monteiro-Riviere, N.A.; Kannan, R.; Riviere, J.E. A computational framework for interspecies pharmacokinetics, exposure and toxicity assessment of gold nanoparticles. *Nanomedicine* **2016**, *11*, 107-119.
36. Lu, X.-F.; Bi, K.; Chen, X. Physiologically based pharmacokinetic model of docetaxel and interspecies scaling: comparison of simple injection with folate receptor-targeting amphiphilic copolymer-modified liposomes. *Xenobiotica* **2016**, *46*, 1093-1104.
37. MacCalman, L.; CL, T.; Kuempel, E. Development of a bio-mathematical model in rats to describe clearance, retention and translocation of inhaled nano particles throughout the body. In Proceedings of the Journal of Physics: Conference Series, **2009**; p. 012028.

38. Mager, D.E.; Mody, V.; Xu, C.; Forrest, A.; Lesniak, W.G.; Nigavekar, S.S.; Kariapper, M.T.; Minc, L.; Khan, M.K.; Balogh, L.P. Physiologically based pharmacokinetic model for composite nanodevices: effect of charge and size on in vivo disposition. *Pharmaceutical research* **2012**, *29*, 2534-2542.
39. Opitz, A.W.; Wickstrom, E.; Thakur, M.L.; Wagner, N.J. Physiologically based pharmacokinetics of molecular imaging nanoparticles for mRNA detection determined in tumor-bearing mice. *Oligonucleotides* **2010**, *20*, 117-125.
40. Perazzolo, S.; Shen, D.D.; Ho, R.J. Physiologically Based Pharmacokinetic Modeling of 3 HIV Drugs in Combination and the Role of Lymphatic System after Subcutaneous Dosing. Part 2: Model for the Drug-combination Nanoparticles. *Journal of Pharmaceutical Sciences* **2022**, *111*, 825-837.
41. Pery, A.R.; Brochot, C.; Hoet, P.H.; Nemmar, A.; Bois, F.Y. Development of a physiologically based kinetic model for 99 m-technetium-labelled carbon nanoparticles inhaled by humans. *Inhalation Toxicology* **2009**, *21*, 1099-1107.
42. Rajoli, R.K.; Back, D.J.; Rannard, S.; Freel Meyers, C.L.; Flexner, C.; Owen, A.; Siccardi, M. Physiologically based pharmacokinetic modelling to inform development of intramuscular long-acting nanoformulations for HIV. *Clinical pharmacokinetics* **2015**, *54*, 639-650.
43. Silva, A.H.; Lima Jr, E.; Mansilla, M.V.; Zysler, R.D.; Pisciotti, M.L.M.; Locatelli, C.; Rajoli, R.K.R.; Owen, A.; Creczynski-Pasa, T.B.; Siccardi, M. A physiologically based pharmacokinetic model to predict the superparamagnetic iron oxide nanoparticles (SPIONs) accumulation in vivo. *European Journal of Nanomedicine* **2017**, *9*, 79-90.
44. Sweeney, L.M.; MacCalman, L.; Haber, L.T.; Kuempel, E.D.; Tran, C.L. Bayesian evaluation of a physiologically-based pharmacokinetic (PBPK) model of long-term kinetics of metal nanoparticles in rats. *Regulatory Toxicology and Pharmacology* **2015**, *73*, 151-163.
45. Tsiros, P.; Cheimarios, N.; Tsoumanis, A.; Jensen, A.Ø.; Melagraki, G.; Lynch, I.; Sarimveis, H.; Afantitis, A. Towards an in silico integrated approach for testing and assessment of nanomaterials: from predicted indoor air concentrations to lung dose and biodistribution. *Environmental Science: Nano* **2022**, *9*, 1282-1297.
46. Wenger, Y.; Schneider II, R.J.; Reddy, G.R.; Kopelman, R.; Jolliet, O.; Philbert, M.A. Tissue distribution and pharmacokinetics of stable polyacrylamide nanoparticles following intravenous injection in the rat. *Toxicology and applied pharmacology* **2011**, *251*, 181-190.
47. Zazo, H.; Colino, C.I.; Gutiérrez-Millán, C.; Cordero, A.A.; Bartneck, M.; Lanao, J.M. Physiologically Based Pharmacokinetic (PBPK) Model of Gold Nanoparticle-Based Drug Delivery System for Stavudine Biodistribution. *Pharmaceutics* **2022**, *14*, 406.
48. Zhang, L.; Su, H.; Wang, H.; Li, Q.; Li, X.; Zhou, C.; Xu, J.; Chai, Y.; Liang, X.; Xiong, L. Tumor chemo-radiotherapy with rod-shaped and spherical gold nano probes: shape and active targeting both matter. *Theranostics* **2019**, *9*, 1893.
49. Mitchell, E.E.L.; Gauthier, J.S. Advanced Continuous Simulation Language (ACSL). *Simulation* **1976**, *25*, 72-78.
50. Marcoline, F.V.; Furth, J.; Nayak, S.; Grabe, M.; Macey, R.I. Berkeley Madonna Version 10–A simulation package for solving mathematical models. *CPT Pharmacometrics & Systems Pharmacology* **2022**, *11(3)*, 290-301.
51. Kolpakov, F.; Akberdin, I.; Kiselev, I.; Kolmykov, S.; Kondrakhin, Y.; Kulyashov, M.; Kutumova, E.; Pintus, S.; Ryabova, A.; Sharipov, R. BioUML—Towards a universal research platform. *Nucleic Acids Research* **2022**, *50(W1)*, W124-W131.
52. Romero, R.M.; Bolger, M.B.; Morningstar-Kywi, N.; Haworth, I.S. Teaching of biopharmaceutics in a drug design course: use of GastroPlus as educational software. *Journal of chemical education* **2020**, *97(8)*, 2212-2220.
53. Bois, F.Y. GNU MCSim: Bayesian statistical inference for SBML-coded systems biology models. *Bioinformatics* **2009**, *25*, 1453-1454.
54. Swift, M.L. GraphPad prism, data analysis, and scientific graphing. *Journal of chemical information and computer sciences* **1997**, *37*, 411-412.
55. Rackauckas, C.; Nie, Q. Differentialequations. DifferentialEquations.jl – A performant and feature-rich ecosystem for solving differential equations in Julia. *Journal of open research software* **2017**, *5*.
56. Schmidt, H.; Jirstrand, M. Systems Biology Toolbox for MATLAB: a computational platform for research in systems biology. *Bioinformatics* **2006**, *22*, 514-515.
57. Marino, D.J. Physiologically based pharmacokinetic modeling using microsoft excel and visual basic for applications. *Toxicology Mechanisms and Methods* **2005**, *15*, 137-154.

58. Afantitis, A.; Melagraki, G.; Isigonis, P.; Tsoumanis, A.; Varsou, D.D.; Valsami-Jones, E.; Papadiamantis, A.; Ellis, L.-J.A.; Sarimveis, H.; Doganis, P. NanoSolveIT Project: Driving nanoinformatics research to develop innovative and integrated tools for in silico nanosafety assessment. *Computational and structural biotechnology journal* **2020**, *18*, 583-602.
59. Bauer, R.J. NONMEM tutorial part I: description of commands and options, with simple examples of population analysis. *CPT: pharmacometrics & systems pharmacology* **2019**, *8*, 525-537.
60. Bauer, R.J. NONMEM tutorial part II: estimation methods and advanced examples. *CPT: pharmacometrics & systems pharmacology* **2019**, *8*, 538-556.
61. Stevenson, K.J. Review of OriginPro 8.5. *Journal of the American chemical society* **2011**, *133*, 5621.
62. Eissing, T.; Kuepfer, L.; Becker, C.; Block, M.; Coboeken, K.; Gaub, T.; Goerlitz, L.; Jaeger, J.; Loosen, R.; Ludewig, B.; Meyer, M.; Niederalt, C.; Sevestre, M.; Siegmund, H.U.; Solodenko, J.; Thelen, K.; Telle, U.; Weiss, W.; Wendl, T.; Willmann, S.; Lippert, J. A computational systems biology software platform for multiscale modeling and simulation: integrating whole-body physiology, disease biology, and molecular reaction networks. *Frontiers in Physiology* **2011**, *2*, 4.
63. Willmann, S.; Lippert, J.; Sevestre, M.; Solodenko, J.; Fois F.; Schmitt W. PK-Sim®: a physiologically based pharmacokinetic 'whole-body' model. *BIOSILICO* **2003**, *1(4)*, 121-124.
64. Giorgi, F.M.; Ceraolo, C.; Mercatelli, D. The R Language: An Engine for Bioinformatics and Data Science. *Life* **2022**, *12*, 648.
65. Jamei, M.; Marciniak, S.; Feng, K.; Barnett, A.; Tucker, G.; Rostami-Hodjegan, A. The Simcyp population-based ADME simulator. *Expert Opinion on Drug Metabolism & Toxicology* **2009**, *5(2)*, 211-223.
66. Jamei, M.; Marciniak, S.; Edwards, D.; Wragg, K.; Feng, K.; Barnett, A.; Rostami-Hodjegan, A. The simcyp population based simulator: architecture, implementation, and quality assurance. *In silico pharmacology* **2013**, *1*, 9.
67. Moore, K. TableCurve 3.0. *Journal of chemical information and computer sciences* **1992**, *32*, 392-392.
68. May, R.A.; Stevenson, K.J. Software Review of UN-SCAN-IT: Graph Digitizing Software. *Journal of the American chemical society* **2008**, *130*, 7516.
